# Prophylactic internal iliac artery balloon occlusion in the management of placenta accreta spectrum disorders: a meta-analysis

**DOI:** 10.61622/rbgo/2025rbgo19

**Published:** 2025-05-04

**Authors:** Nicole dos Santos Pimenta, Ana Clara Felix de Farias Santos, Maírla Marina Ferreira Dias, Gabriela Oliveira Gonçalves Molino, Ana Gabriela Alves Pereira, Pedro Henrique Costa Matos da Silva

**Affiliations:** 1 Universidade Federal do Estado do Rio de Janeiro Rio de Janeiro RJ Brazil Universidade Federal do Estado do Rio de Janeiro, Rio de Janeiro, RJ, Brazil.; 2 Universidade Cidade de São Paulo São Paulo SP Brazil Universidade Cidade de São Paulo, São Paulo, SP, Brazil.; 3 Universidade Federal de Campina Grande Campina Grande PB Brazil Universidade Federal de Campina Grande, Campina Grande, PB, Brazil.; 4 Universidade Federal de Ciências da Saúde de Porto Alegre Porto Alegre RS Brazil Universidade Federal de Ciências da Saúde de Porto Alegre, Porto Alegre, RS, Brazil.; 5 Universidade do Estado de São Paulo São Paulo SP Brazil Universidade do Estado de São Paulo, São Paulo, SP, Brazil.; 6 Universidade Federal de Goiás Departamento de Ginecologia e Obstetrícia Goiânia GO Brazil Departamento de Ginecologia e Obstetrícia, Universidade Federal de Goiás, Goiânia, GO, Brazil.

**Keywords:** Placenta accreta spectrum, Iliac artery, Balloon occlusion

## Abstract

**Objective:**

Placenta accreta spectrum (PAS) describes the failure of placental detachment. PAS is a pregnancy-associated life-threatening condition which increases hemorrhage risk. We evaluated safety and efficacy of internal iliac artery balloon occlusion (IIABOC) on bleeding volume among pregnant women with diagnosis or suspicion of PAS.

**Data source:**

We searched PubMed, Embase and Cochrane databases.

**Study selection:**

Randomized controlled trials (RCTs) and observational studies comparing the efficacy of preoperative prophylactic balloon catheters to a control group with standard care in patients with a prenatal screening of PAS.

**Data collect:**

We computed odds ratio (OR) for binary endpoints and mean difference (MD) for continuous endpoints, with 95% confidence intervals (CIs). We performed random effects models and assessed I^2^ heterogeneity statistics.

**Data synthesis:**

Twenty-four studies were included, of whom 1,023 (51%) received balloons and 983 (49%) did not undergo balloon management. Patients receiving IIABOC had a greater decrease in estimated blood loss (MD −0.33; 95% CI −0.55, 0.11) and increase in operation time (MD 17.21; 95% CI 3.43, 30.99). Apgar score at fifth minute (MD −0.22; 95% CI −0.36,−0.07) significantly decreased. There were no significant differences between groups regarding hysterectomy rates (OR 1.35; 95% CI 0.88, 2.09) and maternal intensive care unit admission (OR 0.81; 95% CI 0.51,1.29).

**Conclusion:**

While IIABOC have demonstrated a significant reduction in estimated blood loss, these findings have not been consistently replicated in RCTs and the surgeon's level of experience must be taken into account since it biases the analysis.

## Introduction

Placenta Accreta Spectrum (PAS), is a pregnancy-associated complication defined as the failure of placental detachment related to uterine abnormalities, presence of fibrosis in the uteroplacental interface and enhanced blood supply.^(1–3)^ PAS is a condition that threatens both maternal and life by increasing the risk of obstetric hemorrhage.^(4)^ Although placenta previa and prior cesarean delivery are known risk factors, more than one third of the cases occur in primiparous women, indicating a diversity in etiology.

Prenatal screening methods include ultrasound or magnetic resonance imaging (MRI), but definitive diagnosis is confirmed through intraoperative findings or histopathology. Typical PAS management involves scheduled cesarean section followed by hysterectomy. Recently, multidisciplinary approaches, including radiologic intervention procedures such as uterine artery embolization, have been adopted for prophylaxis against intraoperative bleeding. In addition, one step conservative surgery may be an alternative which promotes an en bloc resection and uterine reconstruction.^(5)^ Internal iliac artery balloon occlusion (IIABOC) may reduce blood loss, however there is a lack of evidence whether this intervention improves maternal outcomes.^(6)^

Since the publication of prior meta-analyses evaluating the role of IIABOC on bleeding events, subsequent studies have been published.^(7–9)^ Further, the prior meta-analysis by Nankali et al.^(8)^ also focused on placenta previa only cases and did not address balloon management specifically in placenta previa and accreta cases.^(7,8)^ The recent meta-analysis conducted by Chen et al.^(9)^ also included patients with PAS as well those presenting only placenta previa. Furthermore, a significant portion of the study population comprised individuals who exhibited only risk factors, lacking any evidence from ultrasound or MRI.^(9)^ Additionally, the 2021 update to the obstetric care consensus by the American College of Obstetricians and Gynecologists (ACOG) highlighted the controversial potential of this intervention, noting that it reduced obstetric hemorrhage in some, but not all, women.^(10)^

Therefore, we aimed to conduct a systematic review and meta-analysis to compare the efficacy of internal iliac artery balloon occlusion versus no intervention during cesarean section in reducing blood loss and improving secondary outcomes; consequently, minimizing fertility threat by hysterectomy.

## Methods

This meta-analysis was registered in the international prospective register of systematic reviews (PROSPERO), aiming to ensure transparency and reduce the risk of reporting bias. The study was designed in accordance with the Preferred Reporting Items for Systematic Reviews and Meta-analyses (PRISMA) reporting guideline and Meta-analysis Of Observational Studies in Epidemiology (MOOSE) guideline.^(11,12)^

Inclusion in this meta-analysis was restricted to studies meeting all the following eligibility criteria: 1) randomized trials or nonrandomized cohort studies; 2) studies enrolling women diagnosed with placenta accreta spectrum (PAS) disorders; and 3) studies that compared the efficacy of internal iliac artery balloon occlusion with a no intervention control during cesarean section. Studies were excluded based on the following criteria: 1) absence of an intervention or control group; 2) studies with overlapping populations to avoid duplication of data; 3) inclusion of patients not undergoing cesarean delivery; and 4) no outcomes of interest. Furthermore, the search strategy imposed no restrictions on the language of the studies, aiming to encompass the widest possible range of relevant research.

Cochrane, Embase and PubMed databases were systematically searched in January 2023. The search strategy included the terms "placenta accreta", "balloon" and "cesarean section", along with their synonyms or related terms. A comprehensive search strategy is available in supplementary material table 1S. Article selection and data extraction were independently taken by two authors, with any disagreements being resolved through consensus.

Outcomes included maternal outcomes: estimated blood loss (EBL); red blood cell (RBC) units transfused; fresh frozen plasma (FFP) units transfused; length of hospital stay; intensive care unit (ICU) admission; operation time; hysterectomy; relaparotomy; disseminated intravascular coagulation (DIC); hospitalization cost. Neonatal outcomes included: birth weight; Apgar score at 1 minute; Apgar score at 5 minutes; neonatal intensive care unit (NICU) admission. Prespecified sub-analyses were conducted, including data limited to randomized controlled trials (RCTs) and analyses using the propensity score matching (PSM) method.

The risk of bias assessment followed the recommendations of the Cochrane Handbook for Systematic Reviews of Interventions, with the Cochrane Collaboration's tool for assessing the risk of bias in randomized trials (RoB 2) and non-randomized studies were assessed with the Risk of Bias in Non-randomized Studies of Interventions (ROBINS-I).^(13)^ Two authors independently assessed the risk of bias, and disagreements were resolved by consultation with the senior author. Accordingly, "high risk" bias was assigned to studies presenting a high risk of bias on any domain of the RoB 2 tool or some concerns for multiple domains, "some concerns" was assigned to studies presenting some concerns on any domain, and "low risk" of bias, if otherwise. The layout was generated by Robvis.^(14)^ Potential publication bias was assessed through visual inspection of funnel plots and analysis of the control lines.

Binary endpoints treatment effects were compared using odds ratio (OR) with 95% confidence intervals (CIs). Cochran Q test and I^2^ statistics were used to assess for heterogeneity; P values > 0.10 and Higgins and Thompson's I^2^ values > 25% were considered significant for heterogeneity. We applied a DerSimonian and Laird random-effect model for outcomes showing significant heterogeneity. Otherwise, a fixed-effect model was used for endpoints considered to have low heterogeneity. For data handling and conversion, we used the guidelines of the Cochrane Handbook for Systematic Reviews of Interventions, as well as to estimate the sample mean and standard deviation from the sample size, median, range and/or interquartile range.^(15)^

Cochrane Review Manager Software (RevMan 5.4) was used to perform statistical analysis (Nordic Cochrane Centre, The Cochrane Collaboration, Copenhagen, Denmark.

## Results

**Figure 1:**
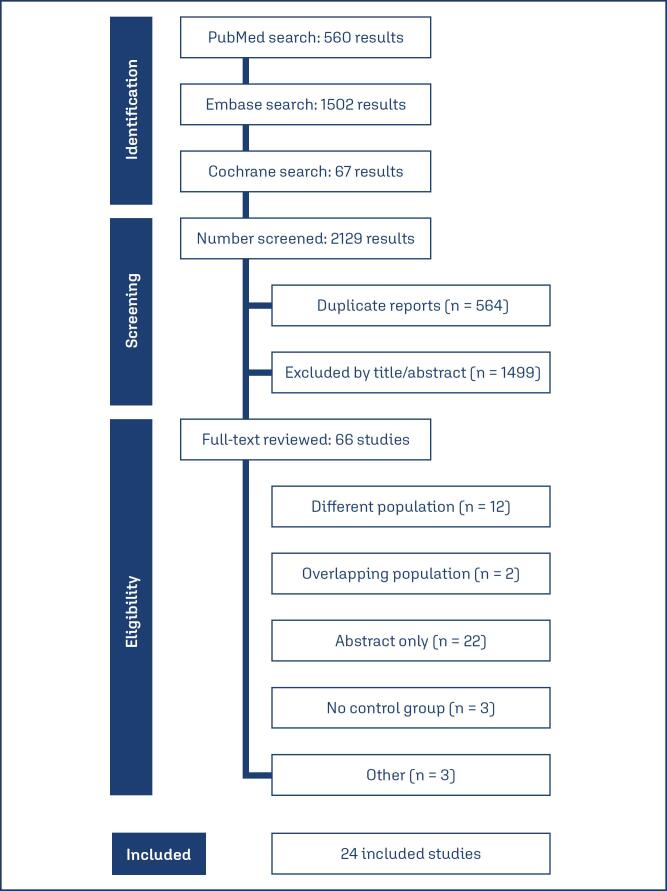
PRISMA flow diagram of study screening and selection

As detailed in figure 1, the initial search yielded 2,129 results. After removal of duplicate records and ineligible studies, 66 remained and were fully reviewed based on inclusion criteria. Of these, 24 studies were included, comprising 2,006 patients from two randomized controlled trials (RCTs), two case-control studies, two prospective cohort studies, and 18 retrospective cohort studies, one of which included a propensity score matching (PSM) analysis. A total of 1,023 (51%) patients received internal iliac artery balloons, while 983 (49%) patients did not undergo balloon management. Most studies utilized ultrasound (US) or histopathology (HP) for the diagnosis or suspicion of PAS. The mean maternal age (MA) ranged from 29.4 to 37.2 years, and parity ranged from 0.9 to 3.7. Hypertensive disorders were reported in 0 to 9 patients, and 0 to 53 women had undergone prior dilation and curettage. The mean number of prior cesarean deliveries ranged from 0.7 to 2. Study characteristics are reported in chart 1.

**Chart 1:** Baseline characteristics of included studies

Study	Study design	N° of patients IIABOC/CG	Method	MA† (y) IIABOC/CG	GA† (wk) IIABOC/CG	BMI† (kg/m^2^) IIABOC/CG	Gravidity† IIABOC/CG	Parity† IIABOC/CG	N° of HDP IIABOC/CG	Prior D&C IIABOC/CG	Prior CD† IIABOC/CG
Bonsen et al. (2023)^(16)^	RCS	22/39	US and IF/HP	36.0/34.0	36.0/37.0^a^	NA	NA	NA	NA	NA	14/27^c^
Cali et al. (2014)^(17)^	PCS	30/23	US and IF/HP	34.2/33.8	243.6/240.6^b^	NA	NA	NA	NA	NA	9/7^c^
Chen et al. (2019)^(18)^	RCS	83/31	US and HP	32.3/31.9	36.9/37.3^a^	27.0/26.6¶	32/15^e^	17/3^f^	3/3	53/20^d^	NA
Chen et al. (2019)^(18)^	RCS and PSM cohort	23/23	US and HP	34.6/32.5	37.0/37.3^a^	27.4/26.4¶	9/11^e^	3/3^f^	1/2	16/14^d^	NA
Chen et al. (2020)^(19)^	RCT	50/50	US/MRI and HP	32.5/33.3	36.4/36.1^a^	26.8/26.6¶	4.0/4.0	1.0/1.0	1/0	2.0/2.0†	44/48^c^
Chen et al. (2021)^(20)^	RCS	248/172	US/MRI and IF/HP	32.5/32.7	36.0/36.0^a^	21.7/21.2	4.0/4.0	1.0/1.0	NA	NA	1.0/1.0†
Cho et al. (2020)^(21)^	RCS	17/25	HP	34.5/34.8	NA	NA	2.2/1.9	1.4/1.3	NA	NA	NA
Fan et al. (2017)^(22)^	PCS	74/89	US/MRI	32.6/32.0	36.5/36.4	26.9/27.2	3.6/3.6	1.1/0.9	2/9	NA	0.9/0.9†
Feng et al. (2017)^(23)^	RCS	30/11	US/MRI	31.0/32.5	259.0/262.0^b^	NA	4.0/4.0	1.0/1.0	NA	NA	NA
Firdous and Aziz (2011)^(24)^	RCS	6/21	US and IF	30.6/29.4	33.5/33.4^a^	NA	NA	NA	NA	NA	NA
Gulino et al. (2018)^(25)^	RCS	16/21	US/MRI and IF/HP	36.2/36.5	34.9/35.1^a^	NA	3.5/3.4	1.2/0.9	NA	NA	0.7/0.8†
Darwish et al. (2014)^(26)^	RCS	32/32	US/MRI and IF/HP	33.8/33.5	35.6/34.3^a^	NA	3.6/3.5	2.0/1.6	NA	NA	NA
Hong et al. (2022)^(27)^	RCS	23/35	US/MRI and HP	33.0/33.3	34.8/35.4	NA	3.7/3.9	2.1/2.1	NA	NA	1.0/1.1†
Li et al. (2018)^(28)^	RCS	37/87	IF/HP	NA/33.4	NA/36.7	NA	NA/0	NA/2.0	NA	NA	NA/1.1†
McGinnis et al. (2019)^(29)^	RCS	12/12	HP	34.0/33.0	33.8/35.0^a^	NA	3.5/3.5	1.9/2.0	NA	0/0†	2.0/1.0†
Mei et al. (2022)^(30)^	RCS	17/15	HP	32.0/34.0	34.4/35.3	NA	4.0/4.0	1.0/1.0	NA	2.0/1.0†	1.0/1.0†
Nieto-Calvache et al. (2021)^(31)^	RCS	30/16	US/MRI	NA	NA	NA	NA	NA	NA	NA	NA
Peng et al. (2020)^(32)^	C/C	48/56	US/MRI	32.0/33.4	35.5/36.0	NA	2.8/3.2	NA	NA	NA	NA
Picel et al. (2018)^(33)^	RCS	90/61	HP	33.3/33.2	33.8/32.4^a^	NA	4.9/5.5	2.9/2.9	NA	NA	24/18^c^
Rosner-Tenerowicz et al. (2021)^(34)^	RCS	15/14	US/MRI and IF/HP	34.0/34.7	35.0/36.0^a^	25.5/28.3	2.0/3.5	NA	NA	NA	1.0/1.0†
Salim et al. (2015)^(35)^	RCT	13/14	US and IF/HP	34.4/37.2	35.1/34.8^a^	26.1/27.2§	4.6/5.4	3.7/3.2	1/4	6/8	2/1^c^
Savukyne et al. (2021)^(36)^	RCS	19/47	US/MRI and HP	33.0/34.0	37.0/37.0	NA	3.0/3.0	2.0/2.0	NA	NA	9/11^c^
Shrivastava et al. (2007)^(37)^	C/C	19/50	US and IF/HP	33.0/34.0	35.3/33.6	NA	NA	NA	NA	NA	5/15^c^
Tan et al. (2007)^(4)^	RCS	11/14	US/MRI	32.0/35.0	36.2/35.7^a^	NA	3.0/5.0	2.0/2.0	NA	NA	1.5/1.4†
Zhou et al. (2021)^(38)^	RCS	58/25	US/MRI and IF/HP	32.3/32.5	35.9/35.8	NA	4.1/4.1	NA	NA	NA	1.0/1.0†

† mean or median;

§ pregestational BMI;

¶ BMI at delivery;

a Gestational age at delivery;

b Gestational age (days);

c One prior CD;

d Two or more prior D&C;

e Five or more;

f Two or more; BMI: body mass index; C/C: case-control; CD: cesarean delivery; CG: control group; D&C: dilation and curettage; GA: gestational age; HDP: hypertensive disorders of pregnancy; HP; histopathology; IF: intraoperative findings; IIABOC: internal iliac artery balloon occlusion group; MA: maternal age; MRI: magnetic resonance imaging; NA: not available; PCS: prospective cohort; PSM: propensity score matching; RCS: retrospective cohort; RCT: randomized controlled trial; US: ultrasound

**Figure 2:**
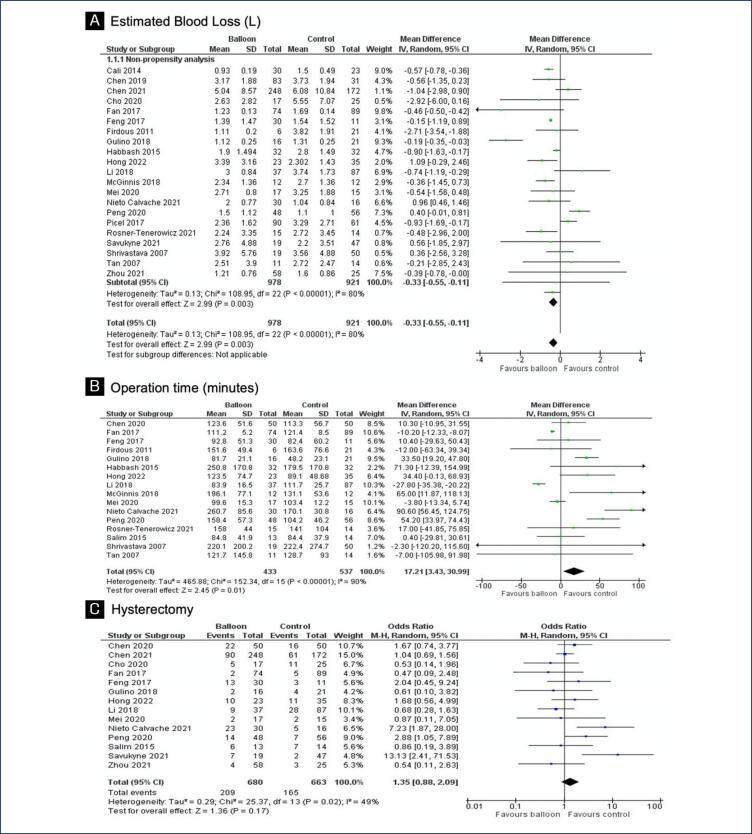
Forest plot of maternal endpoints

**Figure 3:**
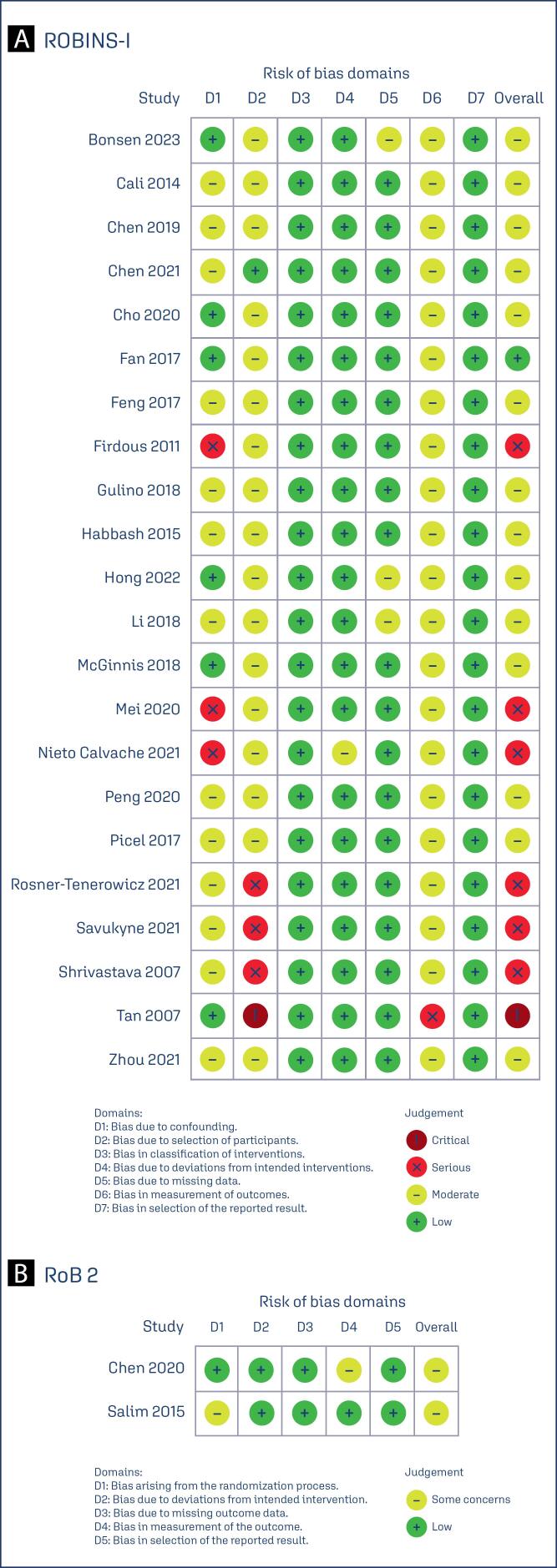
Critical appraisal of individual studies

The primary endpoint analyzed was EBL, which significantly decreased in the intervention group when compared with the control group (MD −0.33; 95% CI - 0.55, - 0.11) (Figure 2A). Balloon occlusion significantly increased operation time (MD 17.21; 95% CI 3.43, 30.99) (Figure 2B). There was no significant difference between the groups regarding hysterectomy incidence (OR 1.35; 95% CI 0.88, 2.09) (Figure 2C). RBC units transfused (MD −0.67; 95% CI −2.06, 0.73), FFP units transfused (MD −0.38; 95% CI −2.18, 1.41), relaparotomy (OR 0.49; 95% CI 0.19, 1.25) and disseminated intravascular coagulation (OR 0.59; 95% CI 0.19, 1.81) represented no statistically significant difference between internal iliac artery balloon and management without balloon (Supplementary Material - Figure 1S). In addition, there was no significant difference in rates of ICU admission (OR 0.81; 95% CI 0.51,1.29), length of hospital stay (MD −0.14; 95% CI −0.76, 0.47) and hospitalization cost (MD 1.91; 95% CI 0.92, 2.89) (Supplementary Material - Figure 2S). Neonatal outcomes as birth weight (MD 0.07; 95% CI −0.04, 0.17), Apgar score at first minute (MD −0.04; 95% CI −0.26, 0.18) and NICU admission (OR 1.02; 95% CI 0.57,1.81) proved to have no statistical significance when comparing study groups. (Supplementary Material - Figure 3S A, B and D). Apgar score at fifth minute (MD −0.22; 95% CI −0.36,−0.07) (Supplementary Material - Figure 3S C) have significantly decreased in the control group when compared with the internal iliac balloon group (Supplementary Material - Chart 1S). All maternal and neonatal outcomes are summarized in supplementary material - chart 2s.

A: Estimated blood loss: non-propensity analysis; B: Operation time; C: Hysterectomy. Cl, confidence interval; MD, mean difference; OD, odds ratio

In the sub analysis of randomized controlled trials (RCT) and propensity score matching (PSM) data involving 173 patients, no significant differences were observed in EBL between the balloon occlusion and control groups (MD 0.02; 95% CI −0.49, 0.53) (Supplementary Material - Figure 4S). Specifically, the comparison between the two groups revealed no significant difference in maternal hemorrhage rates. We performed a leave-one-out sensitivity analysis for EBL outcome. There was a notable decrease in heterogeneity among studies for the outcome of EBL with the removal of and Firdous and Aziz,^(24)^ and Nieto-Calvache et al.,^(31)^ with a reduction from I^2^ = 80% to I^2^ = 73 % (Supplementary Material - Figure 5S). This was likely due to the significant variance in patient characteristics between the groups in Firdous and Aziz^(24)^ as it showed a higher proportion of nulliparous women and previous miscarriage. Nieto-Calvache et al.^(31)^ may have influenced heterogeneity because of its retrospective and observational nature. Overall, there was no significant shift in heterogeneity in the outcomes with the removal of each individual study in the leave-one-out analysis. R software environment, version 4.3.0 (R Foundation for Statistical Computing) was used for sensitivity statistical analysis performance. The risk of bias in individual non-randomized studies is represented by the ROBINS-I traffic-light diagram in figure 3 A. Most studies were assessed as having moderate bias in at least one domain,^(4,15–17,18–33,34–37)^ with seven studies identified as having serious bias in at least one domain.^(23,27,30,33–37)^ A critical judgment was applied to bias due to the selection of participants in one study by Tan et al.^(4)^ For within-randomized study bias, the RoB 2 traffic-light diagram (Figure 3 B) indicates that both RCTs raised some concerns about bias in one domain each.^(18,34,37)^ Additionally, the funnel plot for estimated blood loss demonstrates an asymmetrical distribution of studies with different weights, suggesting significant evidence of publication bias (Supplementary Material - Figure 6S).

A: ROBINS-I; B: RoB 2

## Discussion

In this systematic review and meta-analysis including twenty-four studies and 2,006 patients, internal iliac artery balloon was compared with cesarean only management in patients with placenta accreta spectrum disorders. Internal iliac artery balloon demonstrated the following main findings: (i) a significant reduction in estimated blood loss; (ii) no significant difference in hysterectomy rates; (iii) a significant increase in operation time; (iv) no significance in maternal ICU admission; and (v) a significantly decrease in the Apgar score at fifth minute in control group.

The intervention effectiveness in blood loss decrease remains controversial. The results of our meta-analysis demonstrated that IIABOC promotes a significant decrease in estimated blood loss volume. The significant reductions observed in this outcome with the use of Internal Iliac Artery Balloon Occlusion can be attributed to various reasons. Firstly, the precise positioning of the balloon catheter in the internal iliac artery allows for localized control of bleeding in the pelvis, which is a critical source of significant blood loss during certain surgical procedures.^(39)^ This targeted approach effectively limits the amount of blood flow to the affected area, leading to a reduction in overall blood loss, as well as contributes for a better hysterectomy performance.^(21)^ Additionally, balloon occlusion has demonstrated potent hemostatic effects against arterial bleeding, which may directly contribute to the reduction in this outcome. The temporary blocking of the internal iliac artery can effectively halt bleeding from pelvic fractures, obstetrical hemorrhage, or other sources, thereby reducing the need for extensive blood transfusions and improving patient outcomes.^(45,46)^ Nevertheless, a propensity sub analysis conducted with a smaller patient sample further supported the findings of previous meta-analysis.^(7)^ However, these trials were hampered by limited statistical power because of the relatively small number of patients included.^(17,18,34)^ Another meta-analysis conducted by Chen et al. found similar results, but it is important to interpret them considering the disparities in selection criteria, which led to the inclusion of some studies involving women with only placenta previa or risk factors for PAS without imaging evidence.^(9)^

Massive hemorrhage commonly occurs in PAS disorders as there is not a spontaneous detachment of the placenta from the uterus and, therefore, cesarean hysterectomy may be required for bleeding control.^(40)^ However, if preservation of the patient's fertility is desired and the state is hemodynamically stable, it may be possible to leave the placenta in situ. This approach has several contributions to women's well-being and self-esteem, but it is also important to emphasize the relevance of a continuous long-term monitoring as complications are possible.^(5)^ Given the risks of living the placenta in situ, an one-step conservative surgery may be a better alternative which consists of en bloc resection of the affected myometrium with placenta, succeeded by uterine reconstruction.^(40)^ Furthermore, it is important to analyze the influence IIABOC plays on hysterectomy rates. Our analysis revealed no significant difference between internal iliac artery balloon and standard management.^(41,42)^ This finding is consistent with both included trials, which similarly found no significance concerning cesarean hysterectomy when comparing both intervention and control groups.^(18,34)^

In addition to the emergence of new studies since the publication of previous meta-analyses, as well as the inclusion of women with only placenta previa in Nankali et al.,^(8)^ prior meta-analyses did not analyze the operative duration.^(7,8)^ Individually, most studies revealed no significant difference, however Gulino et al.^(25)^ found a large difference in the operation time, which is associated with a longer duration when using the internal iliac artery balloon. Similarly, our study supports the discovery of a significant increase in surgery duration. It is crucial to highlight that operating time may vary depending on the performance of both radiological and surgical procedures in the same or in different operating rooms, as occurred in Fan et al.^(22,24)^

Maternal intensive care unit admission may be necessary for some patients since hemodynamic status monitoring is important in the postoperative care as there is a risk of abdominal or pelvic bleedings, as well as other complications given the large surgery extent.^(43)^ Ten studies, totalizing 224 women, reported the outcome and our study noted findings that support Liang et al. results.^(7)^ Despite the lower admission incidence in the control group, no statistical difference was found between the two groups. Moreover, ICU admission is important to apply continued vigilance for organ failure and the potential of hypoperfusion.^(10)^

Typically, PAS is associated with higher rates of adverse neonatal endpoints, including a lower Apgar score.^(44)^ Toussia-Cohen et al.^(44)^ described specifically the predominance of an Apgar score lower than seven in the fifth minute in newborns of mothers with PAS. Neonatal outcomes were also analyzed and all but one proved to have no statistical significance. Apgar score at fifth minute had significantly decreased without internal iliac artery balloon management, which demonstrates infant benefits. These findings, though, have not been consistently confirmed by published data found in previous meta-analysis and included studies.^(7,16,20,21,24,26,31,35)^ However, it is valid to emphasize that, despite the Apgar score variation between the two groups, the average of both is above seven.

Placenta accreta spectrum management through internal iliac artery may be related to potential complications. Study data enabled to find no significant difference between balloon and no balloon groups concerning disseminated intravascular coagulation rate.^(26,28,38)^ Although the absence of sufficient data for other surgical complications analysis, it is relevant to highlight possible complications, such as adjacent organ injuries, intrauterine infection and wound infection.^(18,20,23,45,46)^

While this meta-analysis did not find a statistically significant difference in hospitalization costs when comparing internal iliac artery balloon and no balloon, this result should not overshadow that even small cost increases have significant impact on resource allocation and budgeting for healthcare providers.^(47)^ On the other hand, the balloon potential to reduce complications or the need for additional surgical interventions could justify its use despite the higher upfront costs.^(19,47)^ Overall, despite no statistical significance, the resource management and clinical impact of these findings should be considered when deciding for the use of such intervention.

It is essential to interpret our study findings while considering its limitations. Firstly, most studies were non-randomized trials and, although there was a significant sample size, there may be selection bias concerning unblindingness, patients selection, variability in methodology and potential no adequate control for all possible confounding variables. In a propensity analysis using two randomized trials and a propensity cohort, it was possible to analyze the primary outcome with reduced bias. However, these findings may not be consistent as it includes a small sample of women. Secondly, it is also important to highlight the different placental invasion degree distribution in each study as a limitation. Thirdly, the observational and case-control studies inclusion, probably interfered with the heterogeneity increase for some of the outcomes. Lastly, bias concerning the influence of the surgeon's learning curve and level of experience must me taken into account as it influences maternal outcomes.^(30)^

## Conclusion

In conclusion, this meta-analysis has demonstrated that the use of internal iliac artery balloon occlusion (IIABOC) is associated with a decrease in estimated blood loss and an increase in operation time, as well as a decreased in the Apgar score at fifth minute in the control group. Although, no significant results in hysterectomy incidence were found. These findings support that IIABOC may reduce the risk of maternal hemorrhage. However, it cannot yet be recommended as a standard practice. Further analysis and high-quality randomized studies are necessary, as most studies are retrospective and heavily influenced by the surgeon's expertise in PAS surgery.

## Chart 1S Search strategy

**Table:**

Data base	Search strategy	Results
PubMed	("placenta accreta" OR "placenta accrete" OR "placenta prévia") AND ("iliac artery" OR "iliac arteries" OR "internal-iliac artery" OR balloon OR "balloon occlusion" OR "balloon catheter" OR "balloon catheters") AND (cesarean OR caesarean OR "cesarean section" OR "cesarean sections" OR "caesarean section" OR "caesarean sections" OR "c-section" OR "c-sections" OR "c section" OR "c sections" OR hysterectomy OR surgical OR surgery OR perioperative OR intraoperative)	560*
Cochrane	("placenta accreta" OR "placenta accrete" OR "placenta prévia") AND ("iliac artery" OR "iliac arteries" OR "internal-iliac artery" OR balloon OR "balloon occlusion" OR "balloon catheter" OR "balloon catheters") AND (cesarean OR caesarean OR "cesarean section" OR "cesarean sections" OR "caesarean section" OR "caesarean sections" OR "c-section" OR "c-sections" OR "c section" OR "c sections" OR hysterectomy OR surgical OR surgery OR perioperative OR intraoperative)	67*
EMBASE	(‘placenta accreta’/exp OR ‘placenta accreta’ OR ‘placenta accrete’ OR ‘placenta prévia’) AND (‘iliac artery’/exp OR ‘iliac artery’ OR ‘iliac arteries’ OR ‘internal-iliac artery’/exp OR ‘internal-iliac artery’ OR ‘balloon’/exp OR balloon OR ‘balloon occlusion’/exp OR ‘balloon occlusion’ OR ‘balloon catheter’/exp OR ‘balloon catheter’ OR ‘balloon catheters’/exp OR ‘balloon catheters’) AND (cesarean OR caesarean OR ‘cesarean section’/exp OR ‘cesarean section’ OR ‘cesarean sections’ OR ‘caesarean section’/exp OR ‘caesarean section’ OR ‘caesarean sections’ OR ‘c-section’ OR ‘c-sections’ OR ‘c section’ OR ‘c sections’ OR ‘hysterectomy’/exp OR hysterectomy OR surgical OR ‘surgery’/exp OR surgery OR perioperative OR intraoperative)	1502*

* Search strategy performed in January 2024

## Chart 2S Primary and secondary outcomes

**Table:**

Maternal Outcomes				
Outcome	No. of studies	Effect size	95% CI	I^2^
Estimated blood loss* (L)	23	-0.35§	[-0.58, 0.13]	0.32
Estimated blood loss** (L)	3	0.02§	[-0.49, 0.53]	0.320.34
Hysterectomy	14	1.35†	[0.88, 2.09]	0.49
Operating time (min)	16	17.21§	[3.43, 30.99]	0.90
Length of hospital stay (days)	20	-0.14§	[-0.76, 0.47]	0.87
RBC units transfused	13	-0.67§	[-2.06, 0.73]	0.69
ICU admission	10	0.81†	[0.51,1.29]	0.36
Relaparotomy	7	0.49†	[0.19, 1.25]	0.00
FFP units transfused	6	−0.38§	[-2.18, 1.41]	0.49
Hospitalization cost (thousand dollars)	3	1.91§	[0.92, 2.89]	0.88
Disseminated intravascular coagulation	3	0.59†	[0.19, 1.81]	0.00
**Neonatal Outcomes**				
**Outcome**	**No. of studies**	**Effect size**	**95% CI**	**I^2^**
Birth weight (Kg)	12	-0.14§	[-0.32, 0.03]	0.92
Apgar at 1 minute	7	-0.28§	[-0.83, 0.28]	0.83
Apgar at 5 minutes	6	−10.38§	[-45.9 - 25.13]	1.00
NICU admission	5	1.02†	[0.57, 1.81]	0.00

§ mean difference;

† odds ratio;

* non-propensity analysis;

** propensity analysis; CI: confidence interval; FFP: fresh frozen plasma;

I^2^: heterogeneity; ICU: intensive care unit; Kg: kilograms; min: minutes; NICU; neonatal intensive care unit; OR: Odds ratio; RBC: red blood cells
